# A case of primary racemose hemangioma in which the disappearance of an endobronchial lesion was confirmed after bronchial artery embolization

**DOI:** 10.1002/ccr3.3916

**Published:** 2021-02-16

**Authors:** Kazumi Kawabe, Seigo Sasaki, Yuichiro Azuma, Hideya Ono, Tadatoshi Suruda, Yoshiaki Minakata

**Affiliations:** ^1^ Department of Respiratory Medicine National Hospital Organization Wakayama Hospital Hidaka‐gun Wakayama Japan; ^2^ Hashimoto Municipal Hospital Hashimoto Wakayama Japan

**Keywords:** bronchial artery embolization, bronchoscope, hemoptysis, racemose hemangioma

## Abstract

The confirmation of the improvement of endobronchial lesions in addition to that of vascular lesions after bronchial artery embolization of primary racemose hemangioma could be important.

## Graphical abstract

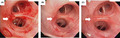

## 1 INTRODUCTION

We experienced a case of primary racemose hemangioma in which an endobronchial lesion was successfully treated by bronchial artery embolization. The case report suggested that the confirmation of the improvement of endobronchial lesions in addition to that of vascular lesions after treatment could be important.

Racemose hemangioma is a rare disease in which the bronchial arteries are markedly bent, meandering, and dilated, which sometimes causes abnormal anastomosis with the pulmonary arteries and veins and the formation of a polypoid lesion in the bronchial lumen. In this paper, we report the case in which the effect of bronchial artery embolization (BAE) on racemose hemangioma was confirmed by the findings of bronchoscopy.

## 2 CASE REPORT

**FIGURE 1:**
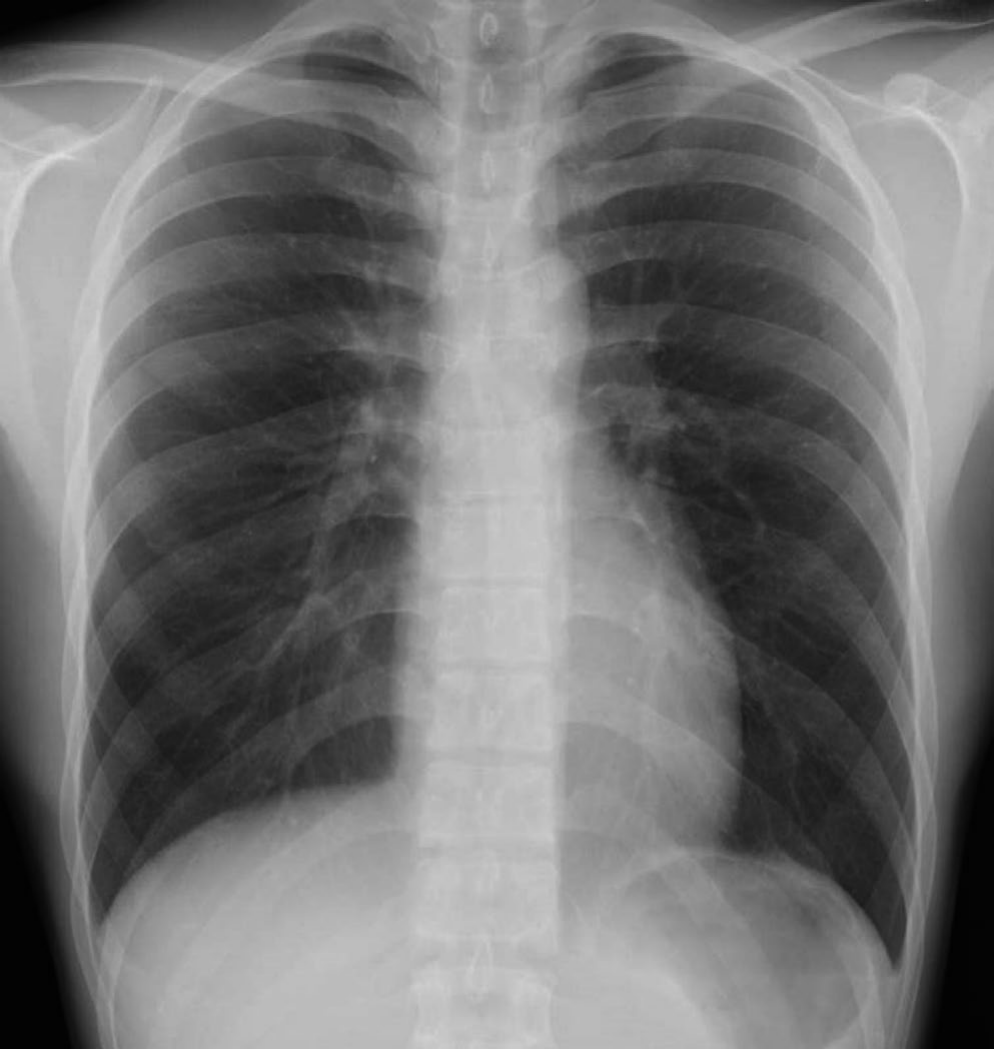
Chest X‐ray at the initial visit. It showed no obvious abnormal findings

**FIGURE 2:**
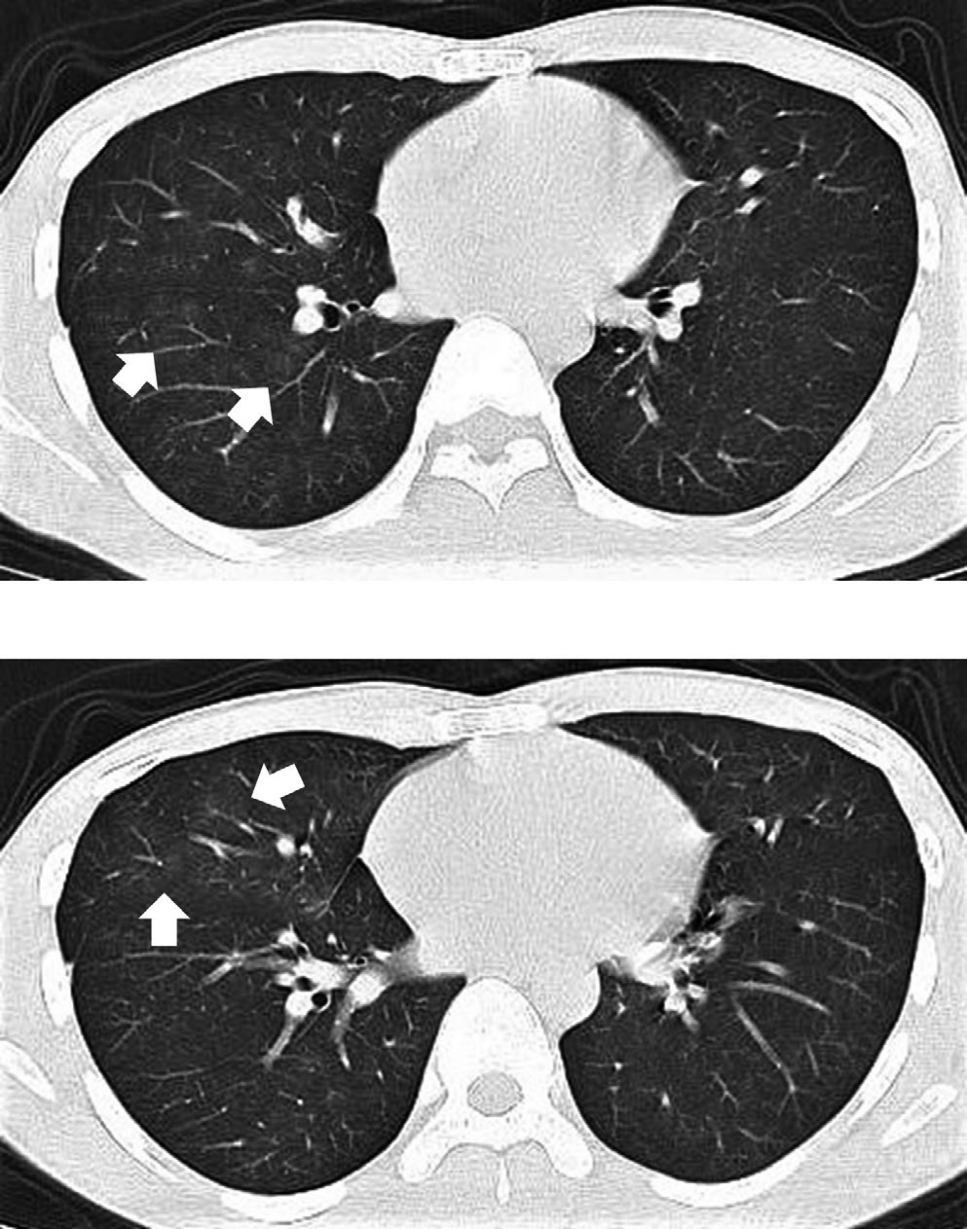
Chest CT at the initial visit. Patchy ground glass shadows (arrow) were observed in the right middle and lower lobe

On 07 February 20xx, a 27‐year‐old man visited a general physician because of a small amount of hemosputum without particular inducement and was prescribed a hemostatic agent. He repeatedly expectorated a small amount of hemosputum, and therefore visited an otolaryngology clinic on 9 February, where blood adhesion was observed under the vocal cords by nasopharyngolaryngoscopy. It was suspected that the bleeding came from the lower respiratory tract, thus, he was introduced to our hospital on 12 February. Chest X‐ray showed no obvious abnormal findings (Figure 1), while Chest computed tomography (CT) revealed patchy ground glass shadows that were suspected to reflect blood inhalation in the right middle and lower lobes (Figure 2). He was hospitalized for examination on 15 February. There were no abnormal laboratory findings (Table 1) with the exception of the chest CT findings.

**TABLE 1:** Laboratory findings at admission

	Result	Reference interval	Unit
Hematology			
White‐blood‐cell count	8.0	3.5‐8.5	10^3^/μL
Red‐blood‐cell count	5.33	4.3‐5.7	10^6^/μL
Hemoglobin	15.1	13.5‐17.0	g/dL
Hematocrit	45.4	40‐50	%
Platelet	193	150‐350	10^3^/μL
Biochemistry			
Aspartate aminotransferase	16	13‐33	IU/L
Alanine aminotransferase	16	8‐42	IU/L
Lactate dehydrogenase	168	119‐229	U/L
Gamma‐glutamyltranspeptidase	17	10‐47	IU/L
Total protein	7.2	6.3‐8.0	g/dL
Albumin	4.5	3.8‐5.3	g/dL
Total bilirubin	1.08	0.30‐1.20	mg/dL
Total cholesterol	206	128‐219	mg/dL
Triglyceride	66	30‐149	mg/dL
Uric acid	4.2	3.6‐7.0	mg/dL
Blood urea nitrogen	10.0	8.0‐22.0	mg/dL
Creatinine	0.87	0.60‐1.10	mg/dL
Fasting blood sugar	90	80‐112	mg/dL
Hemoglobin A1c (NGSP^a^)	5.5	4.6‐6.2	%
C‐reactive protein	<0.01	0.00‐0.30	mg/dL
Carcinoembryonic antigen	1.2	0.0‐5.0	ng/mL
Cytokeratin 19 fragment	1.4	0.0‐3.5	ng/mL
Pro‐gastrin‐releasing peptide	37.8	0.0‐80.9	pg/mL
Quantiferon	(‐)		

^a^ National Glycohemoglobin Standardization Program.

**FIGURE 3:**
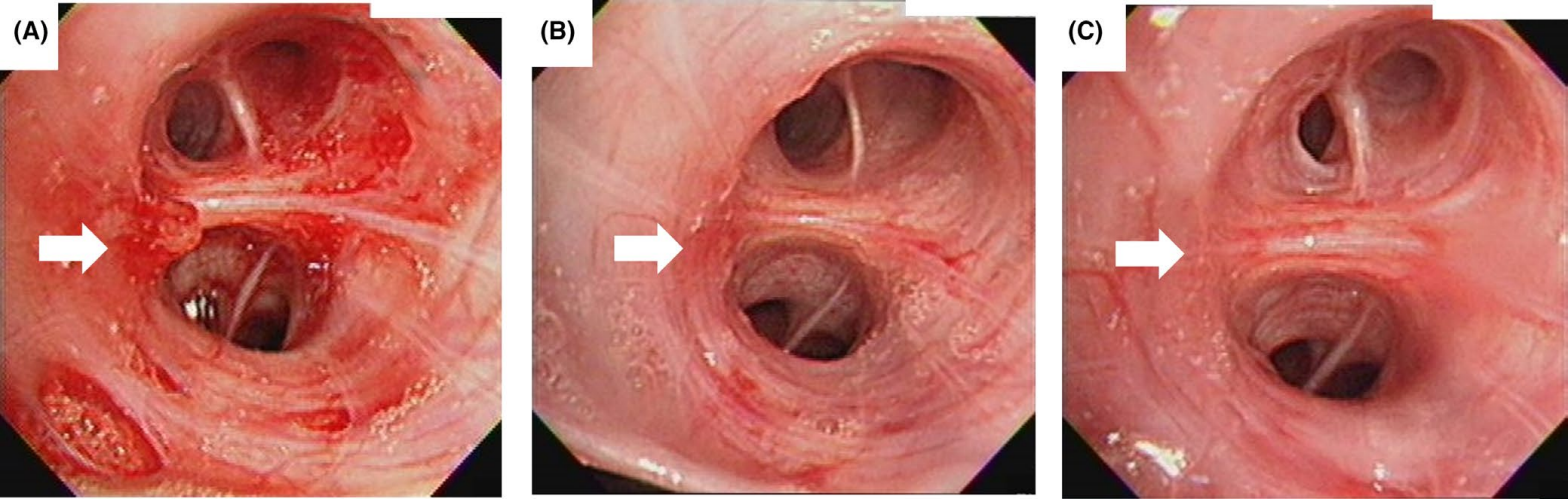
Endobronchial findings. A, Pretreatment. A polypoid lesion (arrow) at the entrance of the right B6 bronchus was observed. B, 4 d after treatment. Mild redness (arrow) of the mucosa remained. C, 10 mo after treatment. The mucous membrane improved (arrow), redness was no longer apparent

Bronchoscopy revealed fresh blood adhered to the entrance of the right B6 bronchus, and a polypoid lesion was observed after the removal of the blood. The pulsation of the lesion was unclear, but it easily bled after only slight contact with the fiberscope (Figure 3A). He was diagnosed with primary racemose hemangioma.

**FIGURE 4:**
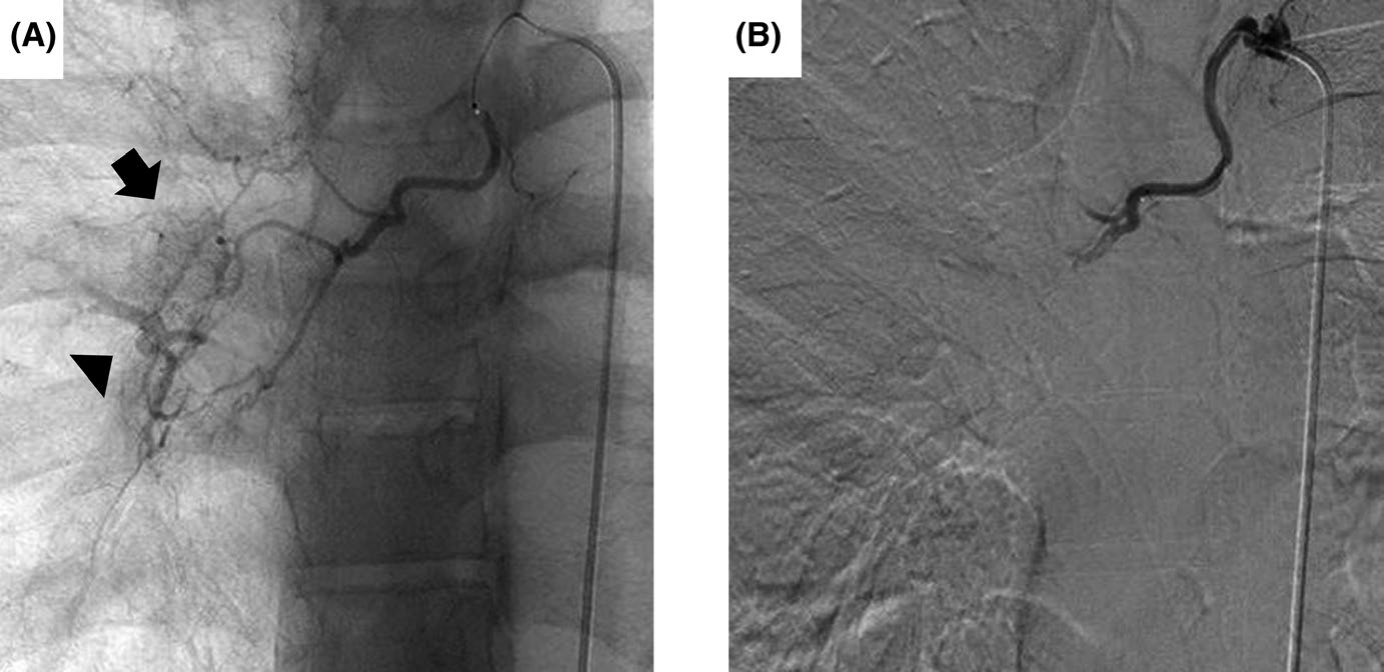
Bronchial arteriography. A, Pretreatment. Abnormal staining (arrow) of the bronchial arteries and shunting (arrowhead) between the bronchial arteries and pulmonary artery were observed. B, Immediately after bronchial artery embolization. Abnormal deep staining of the blood vessels disappeared

While receiving a hemostatic agent by intravenous injection, he was transferred to the Department of Radiology, Wakayama Medical University Hospital on 18 February. He was treated with BAE on the same day. Before BAE, abnormal dark staining and bronchial artery‐pulmonary artery shunt were observed on right bronchial arteriography (Figure 4A). The middle and lower branches of the right bronchial artery were embolized with a gelatin sponge (Serescue^®^, Astellas Pharma Inc). After BAE, the disappearance of abnormal deep staining of the blood vessels was confirmed (Figure 4B).

He was transferred to our hospital on 22 February and received bronchoscopy on the same day. The polypoid lesion at the entrance to the right B6 bronchus had disappeared, and only mild redness of the mucosa remained (Figure 3B). He was discharged on 23 February and was followed at our outpatient clinic. The patient did not experience any subjective symptoms during follow‐up. At 10 months after discharge, bronchoscopy was performed again to observe the endobronchial condition of the bleeding site. On this examination, no polypoid lesion was found and the mucous membrane improved without redness (Figure 3C).

## 3 DISCUSSION

Racemose hemangioma is a very rare disease first reported by Babo et al^1^ in 1976. It is a disease in which the bronchial arteries are remarkably bent, meandering, and dilated, and sometimes show abnormal anastomosis with the pulmonary artery and vein. Similar conditions have also been reported as pulmonary arteriovenous malformation,^2^ bronchial arteriovenous malformation,^3^ bronchial artery aneurysm with associated bronchial artery to pulmonary artery fistula,^4^ and Dieulafoy's disease,^5^, ^6^ however, there is no established definition of the disease at the present time.

This disease is classified as primary due to congenital angiogenesis without bronchial or pulmonary lesions, and secondary due to inflammation such as bronchiectasis and tuberculosis or neoplastic disease.^7^ In the current case, there were no underlying diseases such as pulmonary tuberculosis or bronchiectasis, and no inflammatory findings were found. We, therefore, diagnosed the patient with primary racemose hemangioma.

Most cases of racemose hemangioma are found due to symptoms such as hemosputum, hemoptysis, or dyspnea,^1^, ^2^, ^4^, ^5^, ^6^, ^8^ however, in recent reports, the number of cases found due to asymptomatic chest abnormalities or during examinations for other diseases has increased.^9^, ^10^, ^11^, ^12^ The polypoid lesions in the bronchus are considered to have developed from the dilated bronchial artery.

In the 1980s, most cases of racemose hemangioma were surgically treated,^8^, ^13^ however, since the 1990s, the performance of BAE has increased as a faster and less invasive treatment.^5^, ^8^, ^13^ BAE for hemoptysis was originally reported in 1974 by Remy et al,^14^ and in Japan, it was first reported by Katoh et al^15^ for 33 patients complaining of hemoptysis due to non‐neoplastic disease in 1990. Although BAE is effective as an emergency treatment for hemoptysis, in cases with repeated hemoptysis due to the development of collateral circulation or dilation of blood vessels, it cannot achieve radical hemostasis. Thus, lung lobectomy has been performed, even recently, for these patients, those with massive hemoptysis, and those considered to be high‐risk.^5^, ^6^, ^16^, ^17^ On the other hand, in cases with massive hemoptysis without a shunt, BAE has been reported to be effective.^13^

The efficacy of BAE has been evaluated by the improvement of symptoms or the findings of bronchial arteriography but has not been evaluated based on endobronchial findings. We confirmed the disappearance of the polypoid lesion on the bronchial mucosa at 4 days after or 10 months after BAE treatment. The confirmation of the disappearance of the polypoid lesion that was considered to be a dilated and meandering bronchial artery was direct evidence of the control of the arterial abnormality and risk of re‐bleeding. We, therefore, recommend the observation of endobronchial findings patients with racemose hemangioma who are treated with BAE.

## 4 CONCLUSION

We experienced a case of primary racemose hemangioma in which an endobronchial lesion was successfully treated by BAE. The present case suggested that the confirmation of the improvement of endobronchial lesions in addition to that of vascular lesions after treatment could be important.

## CONFLICT OF INTEREST

The authors declare no conflicts of interest.

## AUTHOR CONTRIBUTIONS

KK: engaged in the treatment of the patient. KK and YM: were major contributor in writing the manuscript. KK, SS, YA, HO, TS, and YM: read, revised, and approved the final manuscript.

## CONSENT

Written consent from the patient has been obtained.

## Data Availability

The data that support the findings of this study are available from the corresponding author upon reasonable request.
